# Use of attention deficit hyperactivity disorder medication among Danish children and adolescents from 2010-2020

**DOI:** 10.1192/j.eurpsy.2025.843

**Published:** 2025-08-26

**Authors:** M. H. Stoltz-Andersen, M. Ernst, S. Dalsgaard, R. T. Wesselhøft, L. Rasmussen

**Affiliations:** 1 Research Unit of Child and Adolescent Mental Health, Mental Health Services in the Region of Southern Denmark; 2 Clinical Pharmacology, Pharmacy and Environmental Medicine, Department of Public Health, University of Southern Denmark, Odense; 3 Center for Child and Adolescent Psychiatry, the Mental Health Services of Capital Region, Glostrup Hospital, Glostrup; 4 Department of Clinical Medicine, Faculty of Health and Medical Science, University of Copenhagen, Copenhagen; 5 National Center for Register-based Research, Business and Social Sciences, Aarhus University, Aarhus, Denmark

## Abstract

**Introduction:**

To ensure rational drug use, there is a need to continously monitor the use of medication for attention deficit hyperactivity disorder (ADHD) among children and adolescents.

**Objectives:**

The aim was to describe the use of ADHD medication among Danish children and adolescents.

**Methods:**

We used data on filled prescriptions of ADHD medication to Danes aged 5-17 years between 2010 and 2020. We calculated the incidence rate, and prevalence proportion, and described treatment duration, age at initiation, prescriber type, and concurrent use of psychotropic medication. Analyses were stratified by age and sex.

**Results:**

The incidence rate of ADHD medication use followed a u-shaped pattern among boys from 2010-2020. This was most pronounced for boys 10-13-years old, with an incidence rate of 0.62 per 100 person-years in 2010, decreasing to 0.35 in 2013, and rising to 0.59 in 2020. The incidence rate among girls increased continuously from 2010 to 2020. The prevalence proportion increased in girls from 0.65% in 2010 to 1.04% in 2020 and in boys from 2.22% in 2013 to 2.65% in 2020. Girls started ADHD medication later than boys (median age 13 vs 11). Child- and adolescent psychiatrists issued 90% of initial prescriptions in 2010 with an increasing proportion over time. Sixty-four percent of 5-9-year-olds and 43% of 10-13-year-olds were covered by an ADHD prescription after five years compared to 27% of 14-17-year-olds. Approximately 20% users in 2020 had concurrent use of other psychotropic medication.

**Conclusions:**

Use of ADHD medication increased in Denmark from 2010-2020. The steady increase in use among girls likely reflect an increased awareness of ADHD in girls. However, the delayed treatment onset in girls should be a focus of attention.

**Disclosure of Interest:**

None Declared

